# Highly active Ce, Y, La-modified Cu/SiO_2_ catalysts for hydrogenation of methyl acetate to ethanol†

**DOI:** 10.1039/c9ra08780j

**Published:** 2020-02-04

**Authors:** Zhiheng Ren, Muhammad Naeem Younis, Chunshan Li, Zengxi Li, Xiangui Yang, Gongying Wang

**Affiliations:** Chengdu Institute of Organic Chemistry, Chinese Academy of Sciences Chengdu 610041 China yangxg@cioc.ac.cn; National Engineering Laboratory & Technology, University of Chinese Academy of Science Beijing 101408 China; CAS Key Laboratory of Green Process and Engineering, State Key Laboratory of Multiphase Complex Systems, The National Key Laboratory of Clean and Efficient Coking Technology, Beijing Key Laboratory of Ionic Liquids Clean Process, Institute of Process Engineering, Chinese Academy of Sciences Beijing 100190 PR China; Zhengzhou Institute of Emerging Technology Industries Zhengzhou 450000 PR China csli@ipe.ac.cn; School of Chemical Science, University of Chinese Academy of Sciences Beijing 100049 People's Republic of China

## Abstract

Rare earth element (Ce, Y, and La) modified Cu/SiO_2_ catalysts *via* hydrolysis precipitation and impregnation method were fabricated for the vapor-phase hydrogenation of methyl acetate to ethanol. LaO_*x*_ showed the most pronounced promotion in the catalytic tests. After detailed characterizations, *via* N_2_ adsorption–desorption, XRD, N_2_O chemisorption, FTIR, H_2_-TPR, H_2_-TPD, TEM, XPS, and TG/DTA, we found that the addition of promoter LaO_*x*_ can decrease the particle size while in turn, it can increase the dispersion of copper species. The strong interactions between copper and lanthanum atoms alter the surface chemical states of the copper species. This results in the generation of more Cu^+^ species and high *S*_Cu_^+^ values, which are responsible for the excellent activity and stability during hydrogenation. In addition, the content of additive LaO_*x*_ and reaction conditions (reaction temperature and LHSV) were optimized. Then, the long-term stability performance was evaluated over the selected catalyst in contrast with Cu/SiO_2_.

## Abstract 2

Highly active and stable La-modified Cu/SiO_2_ catalysts were fabricated for the vapor-phase hydrogenation of methyl acetate to ethanol.

## Graphical abstract

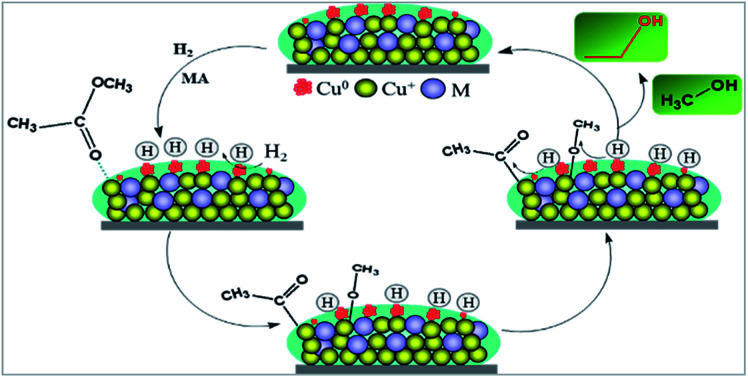

## 1. Introduction

As a renewable and sustainable energy source, ethanol has been widely used as a solvent and disinfectant; it also acts as an alternative fuel for vehicles. At present, a promising approach to produce ethanol from the hydrogenation of dimethyl ether (DME) has been proposed, which has attracted worldwide attention.^1–3^ This process consists of two stages: DME carbonylation to methyl acetate (MA) over zeolites and MA to ethanol over copper based catalysts.^4–8^ The first step acquired higher activity and stability over mordenite, while the second step employed cheap copper catalysts since they possess selective hydrogenation ability.^9,10^

The Cu/SiO_2_ catalyst is typically used for MA hydrogenation mainly because of the non-reducible and relatively inert behavior and low cost of the catalyst, making it suitable for industrial development.^11–13^ Moreover, relatively high copper loading (>30%) is used to synthesize the catalyst to ensure high efficiency of the reaction.^14^ In the main studies on Cu/SiO_2_ catalysts, the proper distribution of Cu species and their strong interactions with the support or promoter are vital in determining the catalytic performance. In the past, different preparation methods have been successfully developed to generate high-efficiency catalysts, including impregnation,^15^ urea-assisted precipitation,^16,17^ ion exchange,^18^ and ammonia evaporation (AE method).^19,20^ Recently, the development of a novel hydrolysis-precipitation method (HP method) by using TEOS as the silicon source exhibited better performance in the hydrogenation of dimethyl oxalate (DMO) to ethylene glycol than that prepared by the AE method.^21^ This could be due to stronger interactions between the copper particles and the silica support, which enhanced the metal dispersion.

The chemical structure, composition, and surface and bulk components greatly influence the properties of the catalysts. In particular, the synergy between Cu^0^ and Cu^+^ species is widely accepted in the hydrogenation of esters. The Cu^0^ active sites are beneficial for activation of H_2_ molecules, while Cu^+^ species could adsorb and activate the C=O groups in MA. The latter is believed to be the rate controlling step.^22,23^ In previous studies, it has been shown that adding a second metal such as Zn, Ni, B, In, Ag, Mn, or Mg to the Cu/SiO_2_ catalyst could improve the catalytic activity.^17,24–29^ The promotion is attributed to the enhancement in copper species dispersion and the appropriate Cu^+^/(Cu^+^ + Cu^0^) ratio on the surface of the catalyst. However, there are still many challenges in Cu-based catalysts that need to be overcome, especially their short lifespan.

Rare-earth metal oxides have been used in many catalytic reactions due to their superb properties, such as enhanced anti-agglomeration and sinterability. Huang *et al.* showed that the rare earth additives (Y, La, Ce, Pr, and Sm) could strengthen the structure of the Cu/SiO_2_ catalyst to improve stability and also prevent the leaching of active metals, especially Y and La.^30^ Zheng *et al.* found that the introduction of La promoter could enhance the interaction between Cu species and silica support, and restrain the sintering of the catalyst in DMO hydrogenation.^31^ Additionally, cerium as a catalyst promoter has exhibited an excellent effect in promoting the hydrogenation of DMO reaction in recent years. Ye *et al.* showed that adding an appropriate amount of cerium could decrease the size of the copper crystallite, improve its dispersion, and enrich the surface Cu^+^ species.^32^ Ai *et al.* found that strong interaction between the Ce promoter and Cu species substantially changed the redox properties of the catalysts. Moreover, the addition of Ce could remarkably increase the dispersion of Cu and retard the sintering of Cu species.^33^ Generally, the introduction of rare-earth metal oxides to Cu/SiO_2_ could increase the copper dispersion and maintain a thermally stable catalyst structure in the hydrogenation reaction. However, to the best of our knowledge, in the hydrogenation reaction of MA, no detailed study on Ce, Y, and La modified Cu/SiO_2_ catalyst system prepared by HP method has been reported.

In this work, a series of Ce-, Y-, and La-promoted Cu/SiO_2_ (Cu-HP) catalysts for the hydrogenation of MA to ethanol were synthesized using the impregnation method. Several characterization techniques were used to evaluate the interaction between copper and the promoters. In addition, the structure–activity relationship of La-doped Cu/SiO_2_ catalyst and the deactivation analysis of Cu/SiO_2_ catalyst were also investigated.

## 2. Experimental

### 2.1 Catalyst preparation

**Fig. 1:**
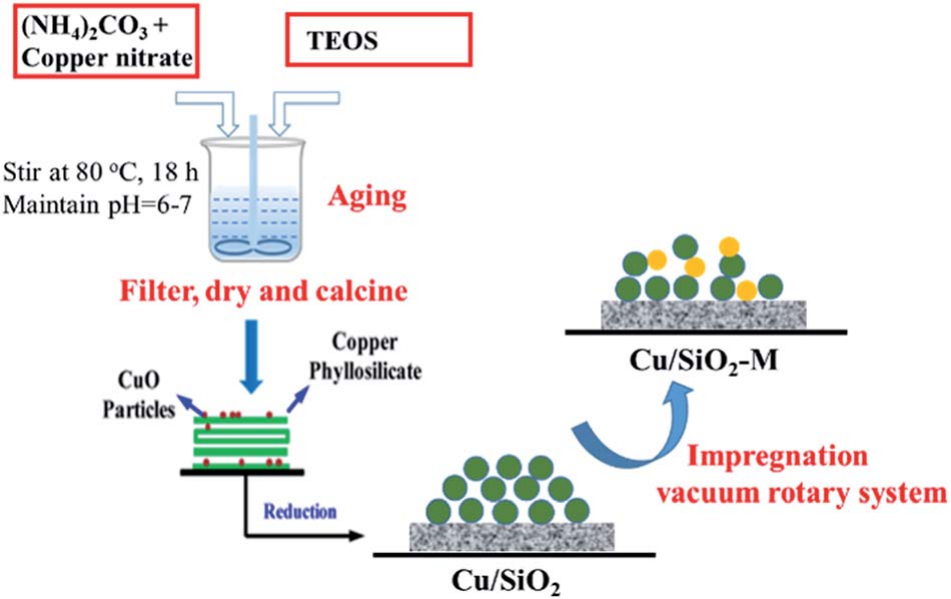
Preparation process of Cu/SiO_2_–*x*M catalysts.

The Cu/SiO_2_ catalyst (30 wt% Cu) was prepared using the hydrolysis precipitation method (HP), as reported previously.^21,24^ Briefly, an appropriate amount of TEOS mixed ethanol solution was first added in the Cu(NO_3_)_3_·3H_2_O aqueous solution and then stirred for 1 h. Subsequently, 0.25 M ammonium carbonate was used as a precipitant and added dropwise to the above solution under vigorous stirring. The pH of the mixed solution was maintained at about 7 and the resultant mixture was aged at 80 °C for 18 h. After filtering, washing, and drying, the dried Cu/SiO_2_ was obtained. Finally, the dried Cu/SiO_2_ was calcined at 500 °C for 4 h in static air and Cu/SiO_2_ powder was obtained. The La, Ce, and Y-modified Cu/SiO_2_ catalysts were prepared by impregnation method. Typically, a certain amount of the above synthesized Cu/SiO_2_ was added to the La(NO_3_)_3_·6H_2_O, Ce(NO_3_)_3_·6H_2_O, and Y(NO_3_)_3_·6H_2_O solution. After stirring for 12 h, excess water was evaporated from the suspension at 80 °C using a vacuum rotary system. The catalyst Cu/SiO_2_–*x*M was finally obtained after calcining at 450 °C for 4 h in air, where M represents three additive elements and *x* (*x* = 1, 5, 10) represents the weight percentage of the rare earth metal M. Here, *x* was calculated according to the equation:
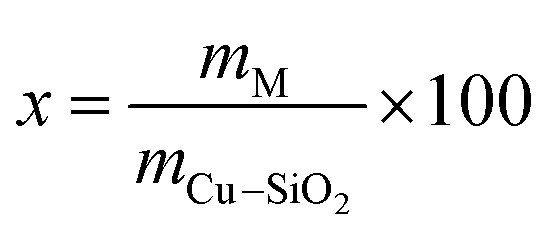


Before testing the reduced catalyst, a certain amount of the Cu-based catalyst was placed into the tube furnace and heated at 350 °C for 4 h under a H_2_ atmosphere (100 mL min^−1^). After cooling to room temperature, the reduced catalyst was sealed in a centrifuge tube to avoid oxidation. The preparation flow chart is shown in Fig. 1. For comparison, a LaO_*x*_/SiO_2_ catalyst was prepared by impregnation using a selected SiO_2_ and the preset weight percentage of La was 5%.

### 2.2 Catalyst characterization

The actual metal loading was determined by ICP-OES on an IRIS Intrepid II XSP (Thermofisher, USA) instrument. X-ray diffraction (XRD) analysis was done on a Rigaku Smart Lab X-ray powder diffractometer using Cu Kα (*λ* = 0.15406 nm) as the radiation source. The data was recorded from 2*θ* = 5° to 90° with a scanning speed at 10° min^−1^. N_2_ adsorption–desorption was performed using a Micromeritics ASAP 2460 analyzer at liquid nitrogen temperature. Prior to testing, all the samples were degassed at 350 °C for 4 h. Fourier Transform infrared spectra (FTIR) was obtained on a Nicolet 6700 spectrometer with a spectral resolution of 4 cm^−1^ and was recorded from 400 to 4000 cm^−1^. KBr was mixed with the sample and then pressed into a wafer. The *in situ* DRIFTs of MA was carried out on the same apparatus as above to identify the nature of the adsorbed species on the catalyst. Prior to testing, the catalyst was firstly reduced at 350 °C in the hydrogenation atmosphere for 4 h. MA was introduced into the cell through the Ar stream. After the catalyst was saturated with MA, the cell was purged with Ar flow and spectra were recorded at different purging times until the spectrum was unchanged. H_2_-TPR curves were recorded to investigate the reduction behavior of the catalyst, which was conducted on an Autochem II 2920 Chemisorption Apparatus (Micromeritics). About 50 mg of the sample was loaded in the U-tube and dried at 120 °C for 1 h under a He atmosphere and then cooled down to 50 °C. 10% H_2_–Ar was switched into the tube and the sample was heated to 500 °C with a ramp rate of 10° min^−1^. H_2_-TPD was measured on the same device above. The sample was first reduced at 350 °C for 4 h in 10% H_2_–Ar atmosphere before cooling down to 50 °C, then the adsorption of H_2_ was performed at 50 °C for 1 h. After the removal of unabsorbed H_2_ by the Ar purge, the desorption data of H_2_ was collected from 50 °C to 800 °C at a heating rate 10 °C min^−1^. The copper surface of the catalyst was calculated in combination with N_2_O-titration and CO-TPD, which was also tested in the above apparatus. As reported previously, it was assumed that Cu^+^ ions and Cu^0^ atoms occupy the same area (1.47 × 10^19^ copper atoms per m^2^).^24,33^ The detailed process is shown in the ESI.† X-ray photoelectron spectroscopy (XPS) and Auger electron spectroscopy (XAES) measurements were performed on an ESCA Lab220i-XL electron spectrometer (VG Scientific) with an Al Kα X-ray radiation source (*hυ* = 1486.6 eV). Before testing, the samples were *ex situ* reduced at 350 °C for 4 h in pure hydrogen and sealed in a centrifuge tube. The C 1s peak (284.6 eV) was used to calibrate the binding energies (BE). TEM micrographs were obtained using the JEM-2100 system. The analysis of elemental dispersion was performed by EDS mapping measurement in the STEM mode. TG and DTA measurements of the spent catalyst were conducted on a simultaneous Shimadzu thermal analyzer. The samples were heated from room temperature to 700 °C in air flow with a heating rate of 10° min^−1^.

### 2.3 Catalyst tests

The catalytic performance of hydrogenation of MA was studied on a fixed bed reactor. A certain catalyst with 40–60 mesh was loaded at the center of the reactor and some silica sand was filled on both sides, to ensure that the feed had a plug flow profile. Prior to the evaluation, all catalysts were pretreated in pure H_2_ flow (100 mL min^−1^) at 350 °C for 4 h, with a ramp rate of 5 °C min. After that, MA was injected into the vaporizer using a high pressure metering pump, heated, and then carried into the reactor by a H_2_ flow. The reaction was conducted at 2.5 MPa, the liquid hourly space velocity (LHSV) was in the range of 1–4 h^−1^, and the gas hourly space velocity (GHSV) was fixed at 3000 h^−1^. The liquid products were separated and analyzed using a Ruihong SP-7890 gas chromatograph equipped with a flame ionization detector (FID). The main by-products were identified by GC-MS (QP2010, Shimadzu, Japan). The internal standard method was used to calculate the conversion of MA and selectivity of ethanol. Iso-butanol was selected as the internal standard material. The conversion of MA, selectivity, and yield of ethanol were based on the following eqn (1)–(3):1
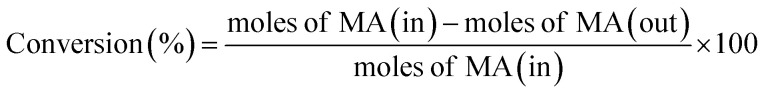
2
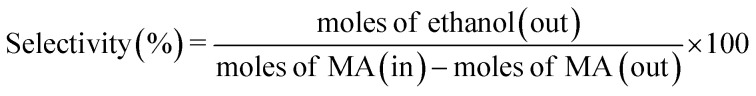
3
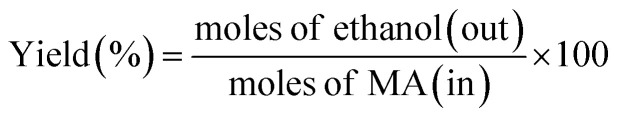


The indicator of space time yield of ethanol (STY) was used to evaluate the catalytic performance of the catalyst. The value of STY_EtOH_ (g_EtOH_ g_cat_^−1^ h^−1^) is the mass of ethanol produced per gram of the catalyst and per hour.

## 3. Results and discussion

### 3.1 Catalytic performance and stability

To investigate the effect of modification by rare earth elements (Ce, Y, and La), the catalytic activities were tested under identical conditions. As shown in Table 1, the MA conversion and ethanol selectivity could be improved by introducing rare earth elements. In addition, under the same amount of additive (5%), the order of activity is Cu/SiO_2_–5La > Cu/SiO_2_–5Y > Cu/SiO_2_–5Ce. For comparison, LaO_*x*_/SiO_2_ was also tested but is not active, indicating a synergistic effect between copper and lanthanum species. Furthermore, the yields of La-doped catalysts exhibit volcano-type trend with increasing La content. The 5% La-doped Cu/SiO_2_ catalyst shows the maximum value of STY (1.04 g_EtOH_ g_cat_^−1^ h^−1^), further increasing the La content to 10%, which decreases to 0.96 g_EtOH_ g_cat_^−1^ h^−1^ be due to partial covering of the active sites.^30^

**Table 1:** Catalytic performance for the hydrogenation of MA over Cu/SiO_2_–*x*M catalysts^a^

Catalyst	MA conversion/%	Ethanol selectivity/%	Yield/%	STY/g_EtOH_ g_cat_^−1^ h^−1^
Cu/SiO_2_	90.4	85.1	76.9	0.89
Cu/SiO_2_–5Ce	92.2	86.2	79.5	0.92
Cu/SiO_2_–5Y	95.1	90.2	85.7	0.99
Cu/SiO_2_–1La	94.7	91.6	86.7	1.00
Cu/SiO_2_–5La	95.4	94.8	90.4	1.04
Cu/SiO_2_–10La	91.5	91.1	83.4	0.96
LaO_*x*_/SiO_2_	0	0	0	0

a Reaction conditions: *T* = 250 °C, *P* = 2.5 MPa, GHSV = 3000 h^−1^, LHSV = 1 h^−1^.

**Fig. 2:**
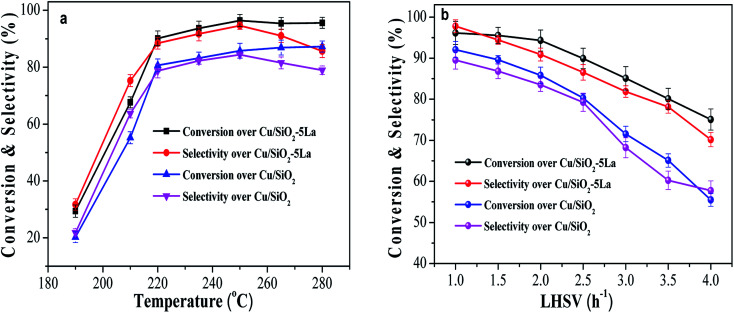
Effect of reaction conditions on the reaction activity. (a) Effect of reaction temperature, *P* = 2.5 MPa, LHSV = 1 h^−1^, GHSV = 3000 h^−1^. (b) Effect of liquid hourly space velocity (LHSV), *T* = 250 °C, *P* = 2.5 MPa, GHSV = 3000 h^−1^.

The different catalytic properties between optimized Cu/SiO_2_–5La and Cu/SiO_2_ were further evaluated as a function of temperature in the range of 190 °C to 280 °C. As shown in Fig. 2a, it is apparent that both conversion and selectivity are improved after La modification. In particular, the La-doping catalyst has a better low-temperature activity. Furthermore, when the reaction temperature is lower than 220 °C, both the conversion of MA (*X*) and the selectivity of ethanol (*S*) sharply rise. On further increasing the temperature, the conversion curves of MA change slightly. However, the selectivity of ethanol for the two catalysts first reaches the maximum (94.6% and 84.4%) at 250 °C and then decreases to 85.6% and 78.9%, respectively. This is because more side reactions occur at higher temperature (280 °C) and the by-product analysis is listed in Table S1.†

The influence of LHSV on the conversion of MA and selectivity of ethanol over Cu/SiO_2_–5La and Cu/SiO_2_ catalysts was tested. As exhibited in Fig. 2b, two catalysts show a relatively high activity under the optimal conditions (LHSV = 1 h^−1^). *X* = 96.1%, *S* = 97.8% were obtained for the Cu/SiO_2_–5La catalyst and *X* = 92.1%, *S* = 89.5% were obtained for the Cu/SiO_2_ catalyst. Excellent performance was obtained at lower LHSV since the catalyst surface possesses enough active sites to adsorb and activate the reactant molecule. As the LHSV increases, the catalytic activity of the two catalysts gradually decline. Because the value of GHSV (dwell time) remain unchanged, the ratio of H_2_/MA will be decreased on increasing the LHSV, which may result in a lower hydrogenation rate. However, for the Cu/SiO_2_–5La catalyst, the conversion and selectivity were maintained above than 90% with an LHSV as high as 2 h^−1^. This suggests the 5% doping La could provide more active species than bare Cu/SiO_2_ catalyst. Moreover, La decorated Cu/SiO_2_ catalyst has a better tolerance capacity for higher LHSV. When the LHSV increases to 4 h^−1^, the conversion and selectivity of Cu/SiO_2_ decrease to 55.5% and 57.8%, respectively. On the other hand, MA conversion and ethanol selectivity of the Cu/SiO_2_–5La catalyst could maintain a better level (75.1% and 70.2%).

**Fig. 3:**
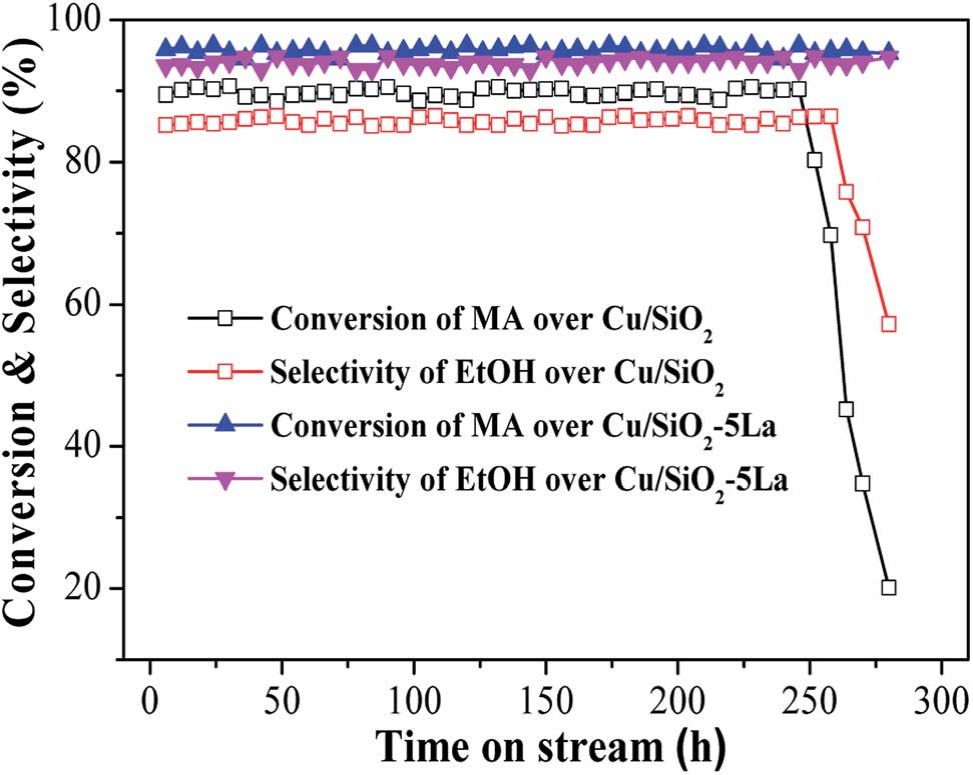
Long-term catalytic performance of Cu/SiO_2_ and Cu/SiO_2_–5La catalysts as a function of reaction time.

In order to investigate the long-term stability of Cu/SiO_2_ and optimized Cu/SiO_2_–5La catalysts, the comparison of catalytic performance was evaluated under the same conditions, *viz.*, *T* = 250 °C, *P* = 2.5 MPa, LHSV = 1 h^−1^, and GHSV = 3000 h^−1^. As displayed in Fig. 3, the two catalysts exhibit good stability within 250 h, indicating that the copper supported SiO_2_ catalysts prepared by the HP method have excellent stability. However, compared with the 5La-doped Cu/SiO_2_ catalyst, MA conversion and ethanol selectivity of the unmodified Cu/SiO_2_ catalyst decreased dramatically after 250 h. The Cu/SiO_2_–5La catalyst could maintain its high activity ever after 280 h, suggesting that modification with a suitable La content could improve the stability of the Cu/SiO_2_ catalyst.

### 3.2 Physicochemical properties of the catalysts

**Fig. 4:**
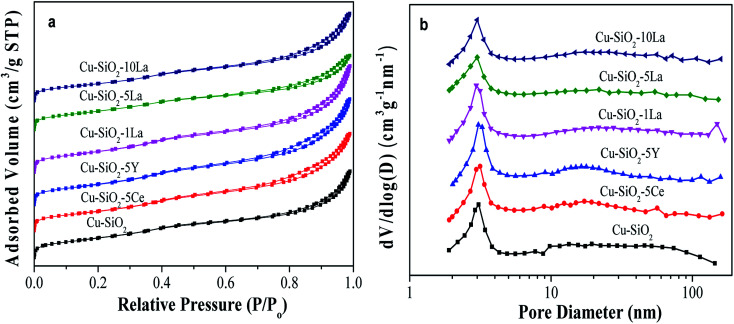
Calcined Cu/SiO_2_–*x*M catalysts (a) N_2_ adsorption–desorption isotherm and (b) BJH pore size distribution curves.

The actual copper loading was measured by ICP-OES. As can be seen from Table 2, this value is slightly lower than the theoretical value due to elution of copper ion during washing. As exhibited in Fig. 4a, all the calcined catalysts show typical IV type isotherms, indicating the existence of a mesoporous structure.^24^ After introducing the rare earth element into the Cu/SiO_2_ system, the shape of the hysteresis loop changes to some extent but the distributions of pore size of all the samples are concentrated at about 3 nm (in Fig. 4b), suggesting that the addition of Ce, Y, and La has no significant change on the pore structure.

**Table 2:** Structural properties of the Cu/SiO_2_–*x*M catalyst

Catalyst	Content^a^ (wt%)		*S*_BET_ b (m^2^ g^−1^)	*V*_p_ b (cm^3^ g^−1^)	*D*_p_ b (nm)	*S* ^0^_Cu_ c (m^2^ g^−1^)	*S*_Cu_^+^ c (m^2^ g^−1^)	*S*_Cu_ (m^2^ g^−1^)
	Cu	M						
Cu/SiO_2_	28.2	—	613	1.16	6.8	28.7	35.0	63.7
Cu/SiO_2_–5Ce	26.6	4.4	575	1.10	7.2	30.1	39.9	70.0
Cu/SiO_2_–5Y	27.0	4.3	596	1.13	7.3	31.5	43.4	74.9
Cu/SiO_2_–1La	25.8	0.99	610	1.14	7.4	26.6	44.8	71.4
Cu/SiO_2_–5La	27.0	4.5	569	0.92	6.9	25.9	46.9	72.8
Cu/SiO_2_–10La	24.2	9.1	522	0.98	7.3	29.4	43.4	72.8
LaO_*x*_/SiO_2_	—	4.3	419	0.99	9.4	—	—	—
SiO_2_	—	—	452	1.06	9.2	—	—	—

a Obtained by ICP-OES.

b Obtained from N_2_ isotherm adsorption.

c Caculated *S*_Cu_ by N_2_O–CO titration combined with LMM XAES spectra.

The BET surface area (*S*_BET_) and pore volume (*V*_p_) of the calcined catalyst are summarized in Table 2. Obviously, the unmodified Cu/SiO_2_ catalyst has a relatively high *S*_BET_ of 613 m^2^ g^−1^ and *V*_p_ of 1.16 cm^3^ g^−1^, which would decrease after introducing the additive may be due to their blockage in the mesoporous structure. Additionally, the distribution of copper species is critical for determining the catalytic activity of the copper based catalyst in the hydrogenation of esters. Therefore, the copper surface areas of the reduced catalysts were estimated by the results of N_2_O titration and combined with CO-TPD and XAES. As shown in Table 2, the Cu^+^ surface area of the reduced sample increases after introducing the promoter, whereas that of the Cu^0^ surface area has no significant difference compared with Cu/SiO_2_. It is worth noting that the *S*_Cu_^+^ values of La-doped Cu/SiO_2_ are higher than the other catalysts, in particular, Cu/SiO_2_–5La has the highest *S*_Cu_^+^ (46.9 m^2^ g^−1^). Nevertheless, as the lanthanum content further increased, the value of *S*_Cu_^+^ is somewhat reduced, which may be due to the coverage of LaO_*x*_ on the catalyst's surface.

### 3.3 Crystalline phase and morphology

**Fig. 5:**
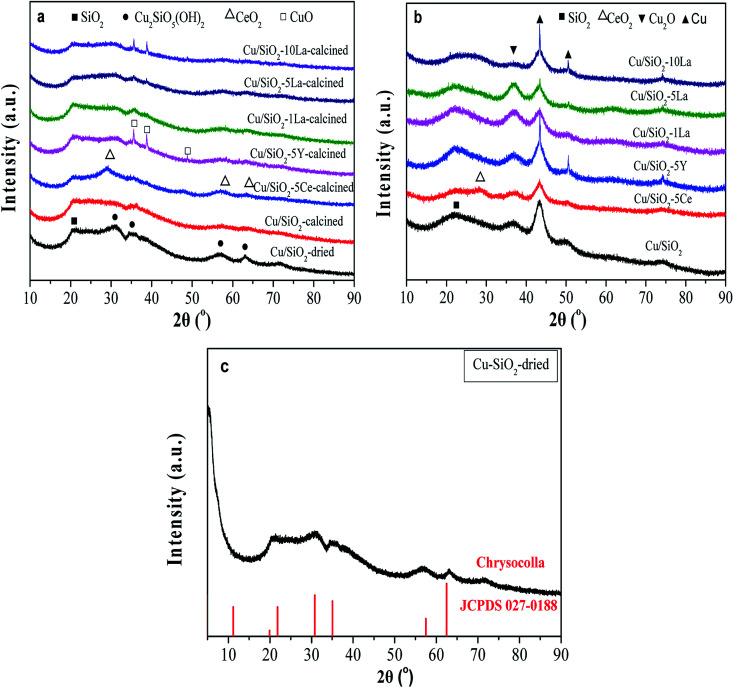
XRD patterns of (a) calcined, (b) reduced Cu–SiO_2_, and Cu/SiO_2_–*x*M catalysts, and (c) the dried Cu/SiO_2_.

As shown in Fig. 5a, the broad diffraction peak that appears at 2*θ* = 22° in the calcined catalyst is assigned to amorphous SiO_2_. For the Cu/SiO_2_ precursor, very faint peaks are ascribable to the Cu_2_SiO_5_(OH)_2_ structure (Fig. 5c).^34^ The structure was also confirmed using FTIR (Fig. S2†), where the band at 670 cm^−1^ is ascribed to the *δ*_OH_ band of Cu_2_SiO_5_(OH)_2_. As could be found, all the calcined Cu/SiO_2_–*x*M catalysts have shown the Cu_2_SiO_5_(OH)_2_ structure. After calcination, the crystallinity weakens, demonstrating that this phase gradually vanishes instead of the emergence of diffraction peaks belonging to the CuO center at 2*θ* = 35.4° and 38.6° (JCPDS05-0661). After doping with the additive of 5% Ce, 1% La, and 5% La, the CuO diffraction peaks are weakly visible. However, for 5% Y and excess La (10%) doped catalysts, the CuO diffraction peaks become more obvious, possibly due to partial aggregation of metallic copper particles. In addition, except for the insignificant diffraction peaks ascribed to CeO_2_ at 28.5° and 56.3° (JCPDS 34-0394), no diffraction peaks assignable to YO_*x*_ or LaO_*x*_ were observed for Y and La modified Cu/SiO_2_ catalysts, respectively. This demonstrates that MO_*x*_ (CeO_2_, YO_*x*_, and LaO_*x*_) are highly dispersed in these samples.

As displayed in Fig. 5b, besides amorphous SiO_2_, other diffraction peaks of the catalysts are observed after reduction in pure H_2_ at 350 °C for 4 h. The characteristic peaks at 2*θ* = 43.3° and 50.5° belong to the Cu^0^ species (JCPDS 04-0836), and the peak center at 2*θ* = 36.4 is ascribable to the Cu_2_O phase (JCPDS 05-0667).^17,33^ On the whole, after doping Ce and La, the diffraction peaks of Cu^0^ species become relatively broad, suggesting that the addition of these promoters could decrease the crystallite size of Cu^0^ and increase the dispersion of Cu^0^ species. The results of grain size of the copper crystallite of different catalysts are shown in Table S2.† It is interesting to note that the sharp diffraction at about 43° in Cu/SiO_2_–5Y and Cu/SiO_2_–10La catalysts might be caused by a small degree of agglomeration of the copper grains. Furthermore, when 5% Y, 1% La, and 5% La are added, the intensity of the Cu_2_O characteristic peak becomes stronger, suggesting that the amount of Cu_2_O is simultaneously increased. However, adding 5% Ce or increasing the La loading to 10% will decrease that strength. These results demonstrate that the Cu/SiO_2_–5La catalyst may have a higher content and a higher dispersion of the Cu_2_O species.

**Fig. 6:**
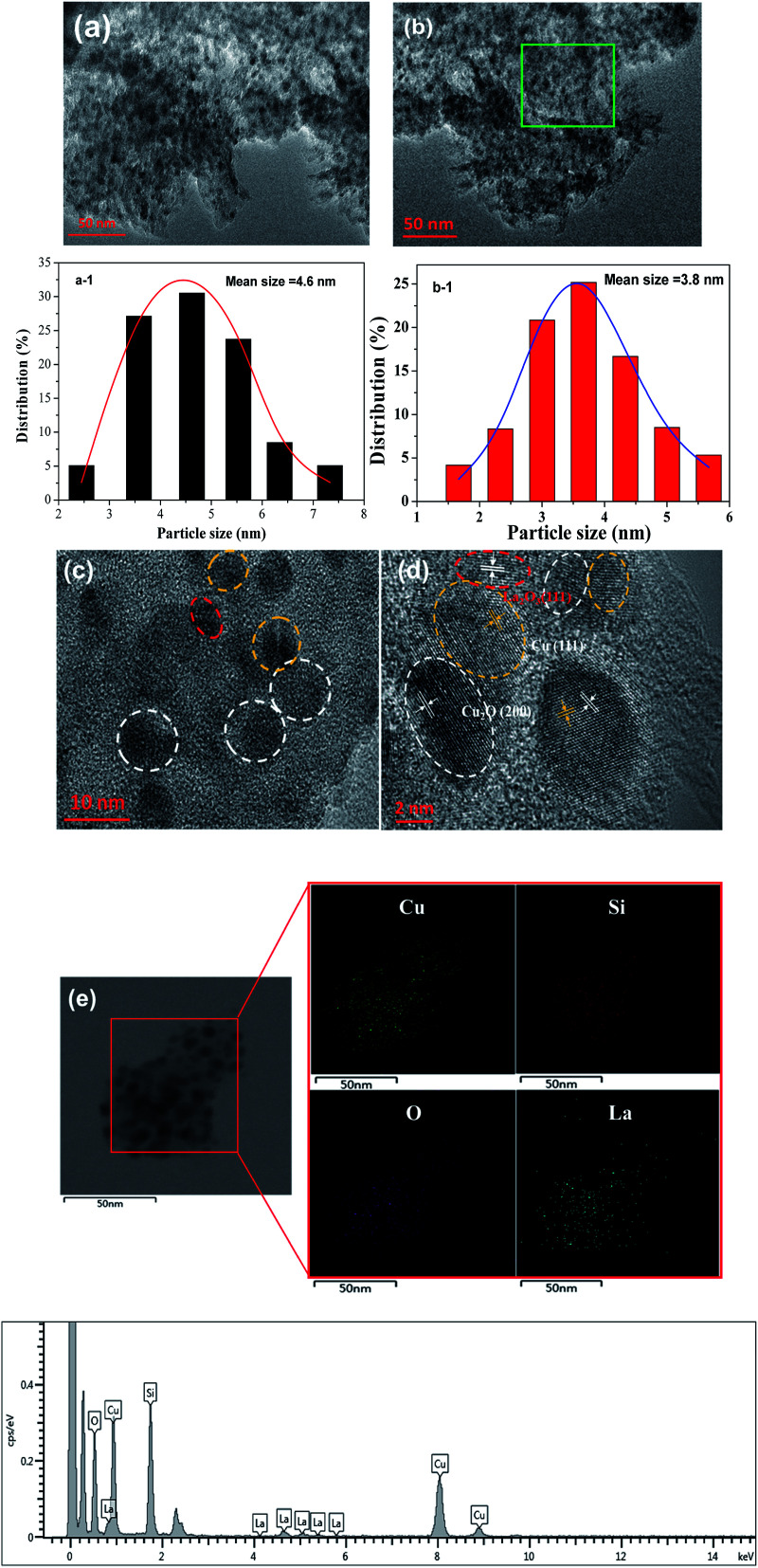
TEM images of reduced (a) Cu/SiO_2_ and (b) Cu/SiO_2_–5La catalyst; particle size distribution of Cu/SiO_2_ (a-1) and Cu/SiO_2_–5La (b-1); (c) and (d) HRTEM images of reduced Cu/SiO_2_–5La catalyst (the zoom-in area of green region); (e) STEM image elemental map of the reduced Cu/SiO_2_–5La catalyst and the corresponding EDS elemental mappings of Cu, Si, O, and La.

HRTEM was used to further determine the morphology and distribution of elements of the optimized Cu/SiO_2_–5La catalyst. As can be seen from Fig. 6a and b, traces of the typical whisker-shaped copper phyllosilicates remain in the reduced Cu/SiO_2_ Cu/SiO_2_–5La catalyst, which are inherited from the structural characteristics of the dried catalyst.^21^ After reduction and activation in pure H_2_ at 350 °C, black metallic Cu particles are formed and are uniformly dispersed on the surface of SiO_2_ texture. The mean particle size is 4.6 nm for the Cu/SiO_2_ catalyst and 3.8 nm for the Cu/SiO_2_–5La catalyst. This means that the addition of La could decrease the particle size of the copper species. In addition, it can be seen from the results of the HRTEM images in Fig. 6c and d that Cu, Cu_2_O, and La_2_O_3_ nanoparticles are present simultaneously in the reduced Cu/SiO_2_–5La catalyst, which is consistent with the XRD results. Furthermore, the HAADF-STEM and STEM-EDS mappings in Fig. 6e show that the Cu and La species are co-existent and uniformly dispersed on the surface of texture of the support, which overlap with each other, suggesting that they are in intimate contact. The strong interaction between Cu and La might be derived from their close proximity, which would retard the transformation of Cu^2+^ to Cu^0^ to some extent.^36^

### 3.4 H_2_-TPR and H_2_-TPD

**Fig. 7:**
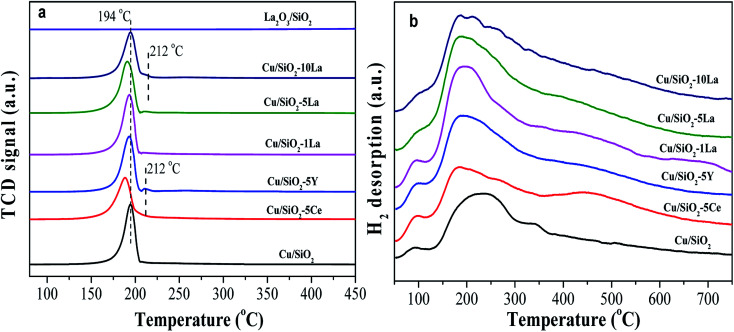
H_2_-TPR profiles (a) of Cu–SiO_2_ and Cu/SiO_2_–*x*M catalysts, (b) H_2_-TPD profiles of reduced Cu–SiO_2_ and Cu/SiO_2_–*x*M catalysts.

To clarify the reducibility of the rare earth element modified Cu/SiO_2_ catalyst, the H_2_-TPR profiles of the calcined catalysts were recorded. As shown in Fig. 7a, all the samples exhibit a center of H_2_ consumption peak at about 194 °C, which could be attributed to the collective effect from highly dispersed CuO to Cu^0^ and copper phyllosilicate to Cu_2_O. The further reduction of Cu_2_O to Cu^0^ requires a high temperature above 600 °C.^37–39^ However, a small H_2_ consumption peak located at 212 °C for 5% Y and 10% La doped Cu/SiO_2_ catalysts may be caused by conversion of large CuO particles to Cu, in accordance with the XRD diffraction peaks of the calcined catalysts in Fig. 5a. After introducing the promoter, the reduction temperature of the catalyst shifts to a lower temperature than that for Cu/SiO_2_, suggesting that a suitable amount of the promoter could prove the dispersion of copper species and be easily reduced.^31^ Moreover, it has been found that the introduction of different contents of La would have a great effect on the reducibility of the catalyst. The reduction peak shifts to lower temperature as the La content is no more than 5%. However, on increasing the La content to 10%, the reduction temperature would shift to a higher temperature due to the significant interaction between the Cu and La species, which is consistent with Huang's results.^27^

H_2_-TPD was used to analyze the adsorption behavior of H_2_ on the catalyst surface after activation in pure H_2_. As presented in Fig. 7b, the H_2_-TPD profiles for all the copper based catalysts show two types of desorption peaks, a lower temperature range (lower than 100 °C), and a broad higher temperature range (150–550 °C). The lower temperature desorption peak is ascribed to the chemisorption of H_2_ at the Cu active sites, while the higher one corresponds to the chemisorbed splitting H species and the broad width of which may be related to the formation of a small size of particles.^40,41^

The strength of a broad high temperature peak can be increased by the introduction of a promoter, suggesting that the addition of a promoter to the Cu/SiO_2_ catalyst can increase the adsorption concentration of the active H species on the catalyst surface. Zheng and Ai believed that the high dispersion of copper species and large *S*_Cu_ was beneficial for the activation and adsorption of H_2_.^31,33^ However, in combination with our results from Table 2, the *S*_Cu_ values of the modified catalysts are almost the same; even the 1% La and 5% La doped catalysts have lower *S*_Cu_ values than other catalysts.

Based on previous studies, there was an electronic effect between copper and the rare earth element, in which the d-electrons of Cu^0^ easily flow to the d orbital of the rare earth element, thereby forming an unoccupied d-orbital and possibly bonding with the H_2_ species.^42,43^ The electrons of Cu–M (M = Ce, Y, and La) catalysts can be transferred to the H_2_ molecule, which promotes dissociation by weakening the H–H bond when H_2_ is adsorbed on the catalyst surface.^44,45^ Therefore, we speculate that the presence of an electronic effect between Cu^0^ and these additive species is the main cause for promoting the adsorption ability of active H species. Among these catalysts, 5% La-doped Cu/SiO_2_ has the maximum adsorption capacity of H species, further increasing the La content; thus, the capacity would be decreased. Therefore, the introduction of an appropriate amount of La species could greatly increase the amount of splitting H species through electronic effects.

### 3.5 Chemical states of the surface species

**Fig. 8:**
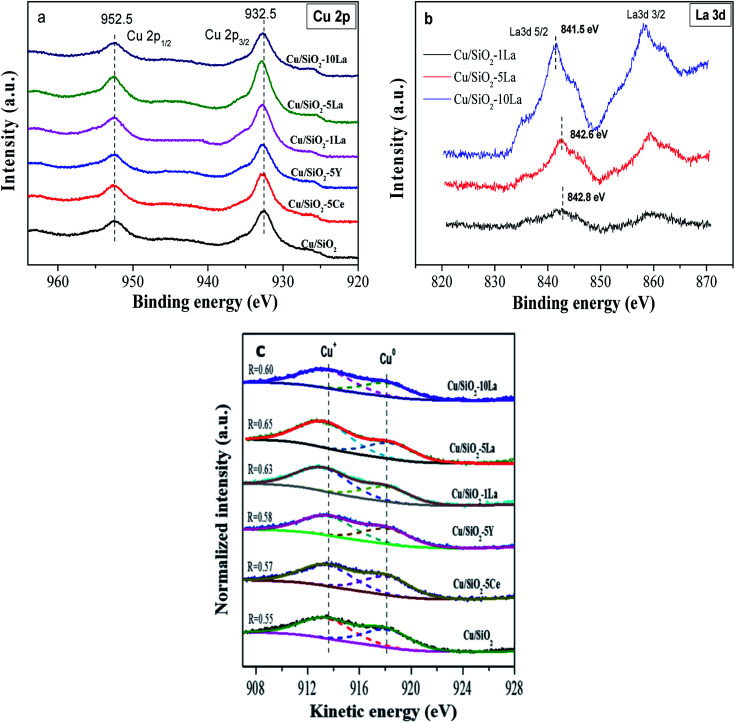
Cu 2p spectra (a), La 3d spectra (b), and (c) Cu LMM Auger spectra of reduced Cu/SiO_2_ and Cu/SiO_2_−*x*M catalysts.

Fig. 8a illustrates the valence state of the copper species on the surface of the reduced catalysts. Apparently, the spectra of the reduced catalyst in our test only have two peaks, which center at 932.6 eV and 952.5 eV corresponding to Cu 2p_3/2_ and Cu 2p_1/2_, respectively. The results demonstrate that the Cu^2+^ species are mostly reduced to Cu^0^ or Cu^+^ since no 2p to 3d satellite peaks between 942–944 eV were observed.^35^ In addition, the binding energy of Cu 2p_3/2_ shifts to higher values after doping with the additive. This confirms the significant electronic effect in the reduced Cu/SiO_2_ catalysts containing Ce, Y, and La, which is consistent with the H_2_-TPD results. The Ce 3d and Y 3d spectra are shown in the ESI† (Fig. S3†). Overall, it could be found that the Ce^3+^ species may be partially present in the reduced Cu/SiO_2_–5Ce catalyst after reduction in our test. It is also worthy of noting that for the La-doped reduced catalysts, the binding energies of La 3d_5/2_ and 3d_3/2_ are centered at 835.5 and 852.1 eV, respectively (Fig. 8b). With the increase in La content, the La 3d_5/2_ binding energy decreases from 842.8 eV to 841.5 eV due to the increase in electron density of the LaO_*x*_ species on the surface of the catalyst.^36^

Cu LMM Auger electron spectra (XAES) was used to discriminate Cu^+^ and Cu^0^ species because these two species are almost located at the same position of binding energy. As shown in Fig. 8c, the Cu^+^ and Cu^0^ species co-exist since the XAES peaks of the reduced catalysts are asymmetric and broad. These peaks could be deconvoluted into two overlapped peaks at *ca.* 913.6 eV and 918.1 eV, which represent Cu^+^ and Cu^0^, respectively.^46^ As summarized in Table 3, it can be seen that the Cu^+^/(Cu^+^ + Cu^0^) molar ratio increases in the Cu/SiO_2_ catalyst, when Ce, Y, and La are doped. As reported previously, the production of more Cu^+^ species may be due to electronic interaction between the copper species and the promoter.^36^ This effect would retard the degree of reduction of Cu^2+^ species and generate more Cu^+^ on the catalyst surface. Compared to Ce and Y, the La-doped Cu/SiO_2_ catalyst exhibits a higher ratio of Cu^+^/(Cu^+^ + Cu^0^), indicating that La is more favorable for increasing the surface concentration of Cu^+^ species. Moreover, the ratio of reduced La-doped catalyst is initially increased to a maximum value of 0.65 when introducing 5% La and then gradually decreased with a further increase in the La loading, which is similar to the results of XRD of the reduced catalysts. The Auger parameters (AP) of Cu^+^ and Cu^0^ are close to the reported values of 1847.0 eV and 1851.0 eV, respectively.^46^

**Table 3:** Deconvolution results of XPS and Cu LMM XAES of Cu/SiO_2_–*x*M catalysts

Catalyst	KE^a^ (eV)		AP^b^ (eV)		Cu 2p_3/2_ BE^c^ (eV)	*X*_Cu_^+^
	Cu^+^	Cu^0^	Cu^+^	Cu^0^		
Cu/SiO_2_	913.6	918.1	1846.1	1850.6	932.5	0.55
Cu/SiO_2_–5Ce	913.6	918.1	1846.2	1850.7	932.6	0.57
Cu/SiO_2_–5Y	913.6	918.1	1846.2	1850.7	932.6	0.58
Cu/SiO_2_–1La	913.6	918.1	1846.2	1850.7	932.6	0.63
Cu/SiO_2_–5La	913.6	918.1	1846.3	1850.8	932.7	0.65
Cu/SiO_2_–10La	913.6	918.1	1846.2	1850.7	932.6	0.60

a Kinetic energy (KE).

b Auger parameter (AP).

c Binding energy (BE).

### 3.6 *In situ* DRIFTs of MA adsorption

**Fig. 9:**
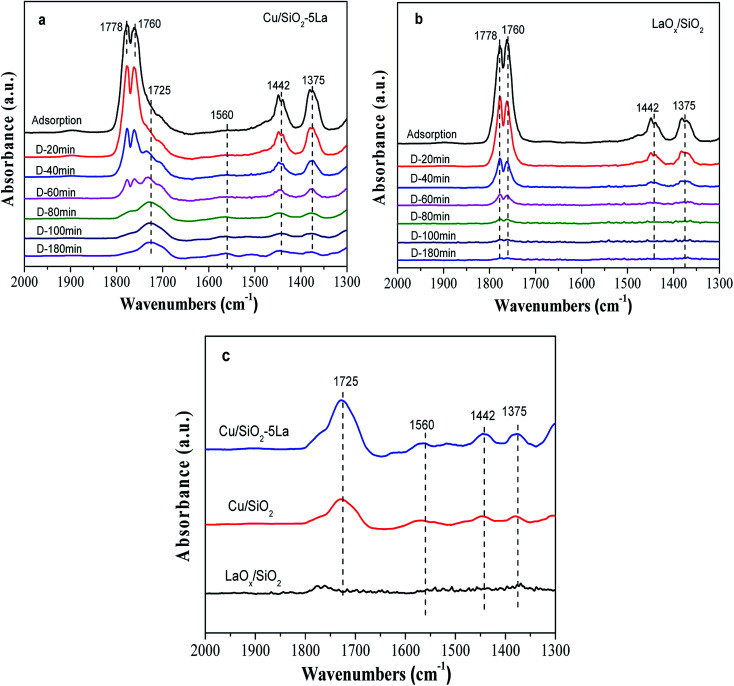
*In situ* DRIFTs of MA adsorption and desorption (*D*-time) of reduced Cu/SiO_2_–5La and LaO_*x*_/SiO_2_: (a) and (b) the spectra during MA desorption process; (c) the final spectra of reduced LaO_*x*_/SiO_2_, Cu/SiO_2_, and Cu/SiO_2_–5La.

In order to affirm the surface species on the catalyst after adsorption of MA and to understand the mechanism of MA hydrogenation, the *in situ* DRIFTs of MA was performed with reduced LaO_*x*_/SiO_2_, Cu/SiO_2_, and Cu/SiO_2_–5La. As shown in Fig. 9a and b, the bands at 1778 cm^−1^ and 1760 cm^−1^ are assigned to the C=O stretching vibration in gaseous MA, and the bands at 1375 cm^−1^ and 1442 cm^−1^ are linked to the symmetrical and asymmetric C–H bending stretching vibrations of acyl species, respectively. This suggests that MA could be adsorbed on the surface of the reduced catalyst or LaO_*x*_/SiO_2_ catalyst and may be partially decomposed into methoxy and acyl species.^47^ The same trend was observed for the intensities of the four bands decrease on prolonging the purging time. However, there is a discrepancy between the adsorption ability of the two catalysts. Compared with the stable adsorption of the reduced Cu/SiO_2_–5La catalyst, the bands at 1375 cm^−1^ and 1442 cm^−1^ of the reduced LaO_*x*_/SiO_2_ catalyst almost disappear after being purged by Ar. This demonstrates that the adsorption of methoxy and acyl species on the reduced LaO_*x*_/SiO_2_ is not stable. In addition, the stretching of *ν*(C=O), shown as the bands at 1725 cm^−1^ and 1560 cm^−1^, is retained in the reduced Cu/SiO_2_–5La catalyst probably due to the strong chemical adsorption of MA.^48^

**Scheme 1:**
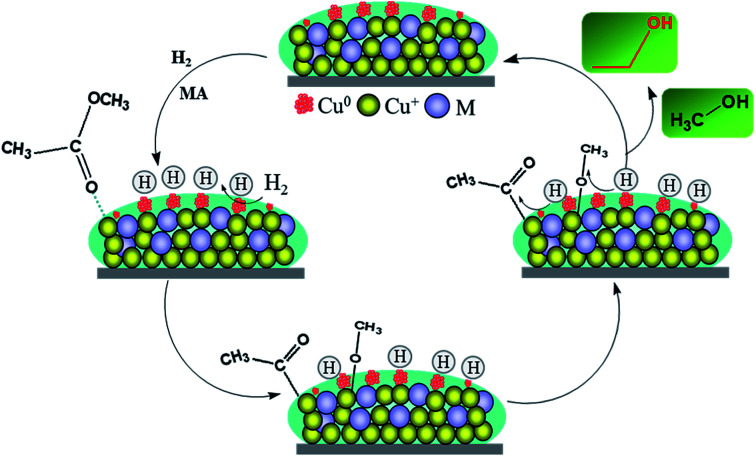
Schematic of MA hydrogenation on the Cu/SiO_2_–La catalyst.

The final spectra of the three catalysts are shown in Fig. 9c. After normalization, the results show that the addition of La could indeed increase the adsorption and dissociation of MA, which is consistent with the XPS results. It could be inferred that the dissociated species (methoxy and acyl species) are mainly adsorbed on the active sites of copper, while the La species themselves may not adsorb these organic species. The adsorption and stabilization of these organics has been proposed to be important in the hydrogenation of esters. In our catalytic system, the addition of a certain La species can create enough Cu^0^ active sites and more Cu^+^ sites, which facilitates the enrichment of surface concentrations of active H-species and organics decomposed from MA. These promotional effects are presumably responsible for the improvement of catalytic performance and stability. Thus, combined with the above analysis, the possible path for the hydrogenation of MA over the Cu/SiO_2_–La catalyst is proposed, as shown in Scheme 1.

### 3.7 Structure–activity relationship

**Fig. 10:**
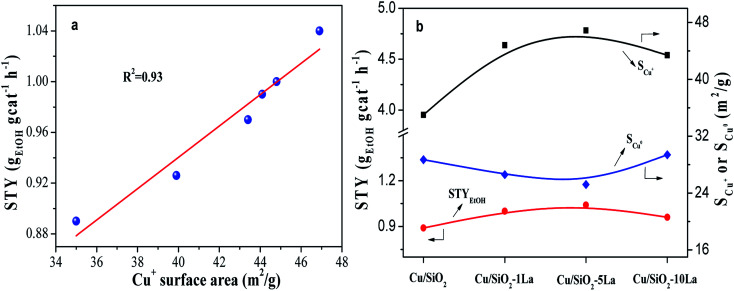
(a) Correlation of STY_EtOH_ and Cu^+^ surface area; (b) effect of La content on STY_EtOH_.

The balanced effect between Cu^0^ and Cu^+^ species is widely accepted for copper-based catalysts in the hydrogenation of esters. Herein, the structure–performance relationship was investigated by correlating the value of STY and Cu^0^ or Cu^+^ species surface area, as shown in Fig. 10a and Fig. S4.† Our results show that there is a direct correlation between STY_EtOH_ and Cu^+^ surface area. However, the relationship between Cu^0^ surface area and STY_EtOH_ is irregular (Fig. S4†). Some researchers have found that more Cu^+^ content on the surface would improve the catalytic activity in hydrogenation esters, such as dimethyl oxalate and MA.^21,24,31^ They pointed out that Cu^+^ species could polarize C=O bond in the esters since they act as electrophilic or Lewis acidic sites, which was the key factor and the rate-controlling step. Moreover, it is noteworthy that Cu/SiO_2_–5La has a large Cu^+^ surface area but the lowest surface area of Cu^0^ (Fig. 10b). Wang *et al.* had reported that the catalytic activity of hydrogenation of MA is linearly increased with increasing Cu^0^ surface area when the available metallic Cu surface was insufficient, otherwise it would be influenced by the Cu^+^ surface area.^22^ Combined with our results, we speculate that the Cu^0^ sites in our prepared catalysts are sufficient to adsorb and activate molecular H_2_; thus, an increase in Cu^+^ concentration leads to heightened catalytic activity.

### 3.8 Comparison of the catalytic activity with various catalysts

As summarized in Table S3,† the MA conversion of Cu-based catalysts prepared using the HP method was superior to the traditional AE method. The AE method used colloidal silica as the silica source and ammonia as the precipitant. In contrast, the HP method used ammonium carbonate as the precipitation agent, which could apparently decrease environmental pollution by reducing ammonia evaporation. Besides, HP method used TEOS as the silica source, which can hydrolyze to generate silanol intermediate in the aqueous solution. This procedure could promote the formation of copper phyllosilicate structure. Thus, the interaction between copper species and support would be enhanced compared with the AE method. Zhao *et al.* have found that the HP method showed higher dispersion and larger surface areas of Cu^0^ and Cu^+^, which was the main reasons for the excellent performance in DMO hydrogenation.^21^ As seen from Table S3,† the Cu/SiO_2_ catalyst prepared by HP method exhibited a better performance (*X* = 86.8%) than that of the catalyst prepared by AE method (*X* = 83.7%) even at a lower reaction pressure.

In comparison to previous literature, the Cu/SiO_2_–5La catalyst shows competitive activity at relatively low ratio of H_2_/MA and low pressure. Therefore, the Cu/SiO_2_–5La catalyst prepared with HP method is a promising new catalyst for the hydrogenation of MA to ethanol.

### 3.9 Deactivation analysis of the Cu/SiO_2_ catalyst

To investigate the deactivation of Cu/SiO_2_ catalyst during the stability test, the carbon depositions of the spent Cu/SiO_2_ catalyst were measured by TG analysis.^49,50^ As shown in Fig. S5,† the carbon deposition of the Cu/SiO_2_ catalyst is not the main cause of deactivation.^51,52^ Moreover, the copper mass loading of the Cu/SiO_2_ and Cu/SiO_2_–5La catalysts after stability testing was measured by ICP. The copper mass loading was slightly decreased from 28.2% to 27.5% for Cu/SiO_2_ and 27% to 26.1% for the Cu/SiO_2_–5La catalyst, respectively. This suggests that the loss of copper species is also not the main factor causing deactivation.

**Fig. 11:**
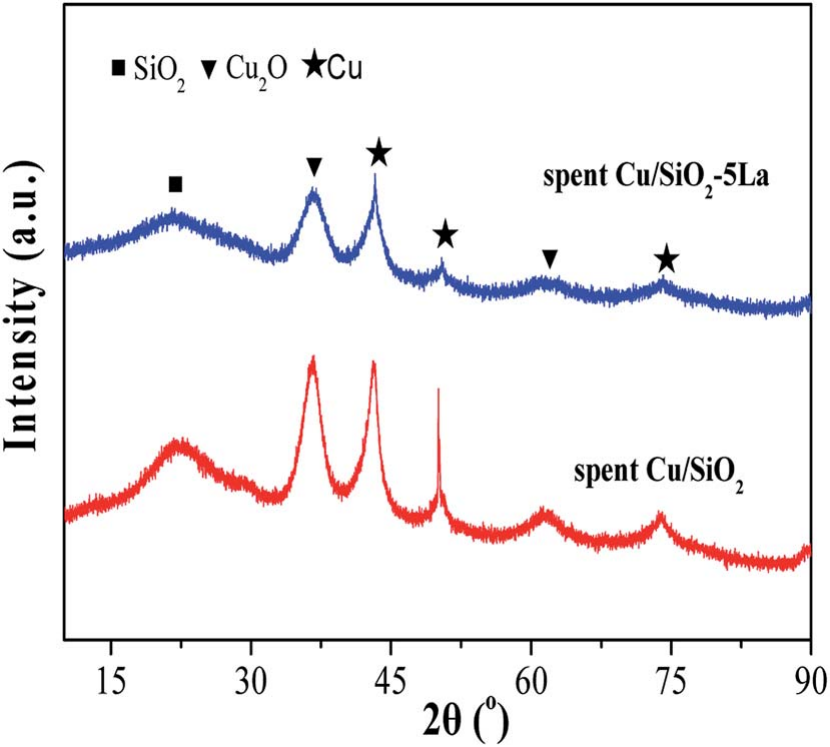
XRD spectra of spent Cu/SiO_2_ after the long-term stability test.

**Fig. 12:**
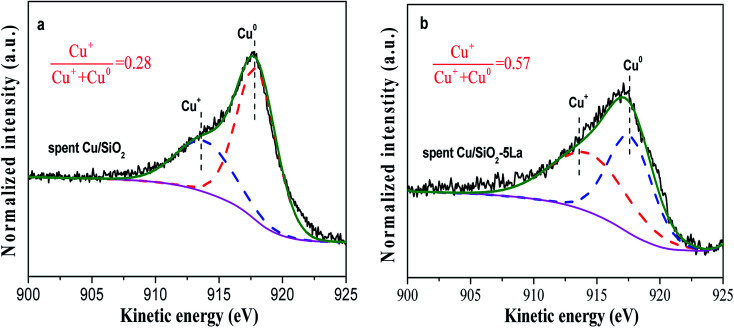
(a) Cu LMM Auger spectra of spent Cu/SiO_2_ and (b) Cu/SiO_2_–5La catalysts.

In general, agglomeration of copper species is believed to be a major cause of deactivation of copper based catalysts.^53,54^ As illustrated in Fig. 11, the XRD analysis of spent Cu/SiO_2_ and Cu/SiO_2_–5La catalysts was also carried out. Compared to the 5% doped Cu/SiO_2_ catalyst, it can be observed that the intensity of the diffraction peak ascribable to metallic Cu^0^ of spent Cu/SiO_2_ become sharper. Compared with the fresh samples, it could be seen that the addition of La could restrain the aggregation of copper. As can be demonstrated from Table S2,† the copper crystallite size of spent Cu/SiO_2_ increased from 4.5 nm to 8.6 nm. From the TEM results of the spent catalysts (Fig. S9†), it could be found that the grain size of the spent Cu/SiO_2_ catalyst after the stability test increased sharply from 4.6 nm to 9.9 nm, which is much larger than that of the spent Cu/SiO_2_–5La catalyst (5.4 nm). Besides, as exhibited in Fig. 12, the Cu^+^/(Cu^+^ + Cu^0^) molar ratio of spent Cu/SiO_2_ declines greatly from 0.55 to 0.28 after long-term evaluation but the 5% La-doped Cu/SiO_2_ catalyst remains relatively stable. Therefore, we speculate that the activity of the Cu/SiO_2_ catalyst after 250 h is greatly reduced due to the concurrent effect of sintering and valence transition of copper species. It can be seen from the above analysis that introducing an appropriate amount of La can not only improve the stability of the original Cu/SiO_2_ catalyst but also modulate the distribution of copper species on the surface of the catalyst, thereby improving the activity and stability under the reaction conditions.

## 4. Conclusion

Three selected rare earth element (Ce, Y, and La) modified Cu/SiO_2_ catalysts exhibited great improvement in the catalytic activity for hydrogenation of methyl acetate to ethanol. Compared to the unmodified Cu/SiO_2_ catalyst (lower MA conversion of 90.4% and ethanol selectivity of 85.1%), the 5% La-doped Cu/SiO_2_ catalyst showed the highest catalytic performance with the MA conversion of 95.4% and ethanol selectivity of 95.8% under the same reaction conditions. There was evidence that strong interactions between copper and lanthanum species can modulate the state of surface copper species. The higher ratio of Cu^+^/(Cu^+^ + Cu^0^) and the corresponding higher Cu^+^ surface area were responsible for the higher value of STY_EtOH_. The Cu^+^ surface area was determined by the content of lanthanum loading. Furthermore, the optimal Cu/SiO_2_–5La catalyst showed excellent long-term stability, which could maintain its initial activity for more than 280 h without any deactivation. The stability may originate from the ability of resistance to sintering and transition of surface copper species by introduction of the lanthanum species.

## Conflicts of interest

The authors declare no competing financial interest.

## Supplementary Material

RA-010-C9RA08780J-s001

## References

[cit1] San X., Zhang Y., Shen W., Tsubaki N. (2009). Energy Fuels.

[cit2] Li X., San X., Zhang Y., Ichii T., Meng M., Tan Y., Tsubaki N. (2010). Direct Synthesis of Ethanol from Dimethyl Ether and Syngas over Combined H-Mordenite and Cu/ZnO Catalysts. ChemSusChem.

[cit3] Jiang Y., Liu Z., Song J., Chang I., Zeng J. (2018). Green Energy & Environment.

[cit4] Cheung P., Bhan A., Sunley G. J., Iglesia E. (2006). Angew. Chem., Int. Ed..

[cit5] Zhou H., Zhu W., Shi L., Liu H., Liu S., Xu S., Ni Y., Liu Y., Li L., Liu Z. (2015). Catal. Sci. Technol..

[cit6] Liu Y., Zhao N., Xian H., Cheng Q., Tan Y., Tsubaki N., Li X. (2015). ACS Appl. Mater. Interfaces.

[cit7] Xue H., Huang X., Ditzel E., Zhan E., Ma M., Shen W. (2013). Ind. Eng. Chem. Res..

[cit8] Nata Santiago M. A., Sıanchez-Castillo M. A., Cortright R. D., Dumesic J. A. (2000). J. Catal..

[cit9] Huang Z., Cui F., Xue J., Zuo J., Chen J., Xia C. (2012). Catal. Today.

[cit10] Brands D. S., Poels E. K., Bliek A. (1999). Appl. Catal., A.

[cit11] Zhong K., Wang X. (2014). Int. J. Hydrog. Energy.

[cit12] Yang D., Sararuk C., Suzuki K., Li Z., Li C. (2016). Chem. Eng. J..

[cit13] Zhao H., Zuo C., Yang D., Li C., Zhang S. (2016). Ind. Eng. Chem. Res..

[cit14] Huang X., Ma M., Miao S., Zheng Y., Chen M., Shen W. (2017). Appl. Catal., A.

[cit15] Yin A., Guo X., Dai W., Li H., Fan K. (2008). Appl. Catal., A.

[cit16] Lin H., Zheng X., He Z., Zheng J., Duan X., Yuan Y. (2012). Appl. Catal., A.

[cit17] He Z., Lin H. Q., He P., Yuan Y. (2011). J. Catal..

[cit18] Yin A., Guo X., Fan K., Dai W. (2010). ChemCatChem.

[cit19] Chen L., Guo P., Qiao M., Run Y., Li H., Shen W., Xu H., Fan K. (2008). J. Catal..

[cit20] Wang Z., Xu Z., Peng S., Zhang M., Lu G., Chen Q., Chen Y., Guo G. (2015). ACS Catal..

[cit21] Zhao Y., Li S., Wang Y., Shan B., Zhang J., Wang S., Ma X. (2017). Chem. Eng. J..

[cit22] Wang Y., Shen Y., Zhao Y., Lv J., Wang S., Ma X. (2015). ACS Catal..

[cit23] Li S., Wang Y., Zhang J., Wang S., Xu Y., Zhao Y., Ma X. (2015). Ind. Eng. Chem. Res..

[cit24] Zhao Y., Shan B., Wang Y., Zhou J., Wang S., Ma X. (2018). Ind. Eng. Chem. Res..

[cit25] Liu H., Huang Z., Kang H., Li X., Xia C., Chen J., Liu H. (2018). Appl. Catal., B.

[cit26] Ye C., Guo C., Sun C., Zhang Y. (2016). RSC Adv..

[cit27] Huang Y., Ariga H., Zheng X., Duan X., Takakusagi S., Asakura K., Yuan Y. (2013). J. Catal..

[cit28] Zhao S., Yue H., Zhao Y., Wang B., Geng Y., Lv J., Wang S., Gong J., Ma X. (2013). J. Catal..

[cit29] Liu Y., Ding J., Sun J., Zhang J., Bi J., Liu K., Kong F., Xiao H., Sun Y., Chen J. (2016). Chem. Commun..

[cit30] Huang Z., Liu H., Cui F., Zuo J., Chen J., Xia C. (2014). Catal. Today.

[cit31] Zheng X., Lin H., Zheng J., Duan X., Yuan Y. (2013). ACS Catal..

[cit32] Ye C., Guo C., Zhang J. (2016). Fuel Process. Technol..

[cit33] Ai P., Tan M., Reubroycharoen P., Wang Y., Feng X., Liu G., Yang G., Tsubaki N. (2018). Catal. Sci. Technol..

[cit34] Yue H., Zhao Y., Zhao S., Wang B., Ma X., Gong J. (2013). Nat. Commun..

[cit35] Gong J., Yue H., Zhao Y., Zhao S., Zhao L., Lv J., Wang S., Ma X. (2012). J. Am. Chem. Soc..

[cit36] Huang J., Ding T., Ma K., Cai J., Sun Z., Tian Y., Jiang Z., Zhang J., Zheng L., Li X. (2018). ChemCatChem.

[cit37] Van Der Grift C. J. G., Wielers A. F. H., Jogh B. P. J., Van Beumun J., De Boer M., Versluijs-Helder M., Geus J. W. (1991). J. Catal..

[cit38] Marchi A. J., Fierro J. L. G., Santamaría J., Monzon A. (1996). Appl. Catal., A.

[cit39] Van Der Grift C. J. G., Wielere A., Mulder A., Geus J. W. (1990). Thermochim. Acta.

[cit40] Dong X., Zhang H., Lin G., Yuan Y., Tsai K. R. (2003). Catal. Lett..

[cit41] Scholten J. J. F., Pijpers A. P., Hustings A. M. L. (1985). Catal. Rev..

[cit42] Huang Z. and Geng J., Industrial Catalysis, Chemical Industry Press, Beijing, 2nd edn, 2006, pp. 85–88

[cit43] Boudjahem A. G., Monteverdi S., Mercy M., Ghanbaja D., Bettahar M. (2002). Catal. Lett..

[cit44] Jun K., Shen W., Rao K. S. R., Lee K. W. (1998). Appl. Catal., A.

[cit45] Zhang L., Zhang Y., Chen S. (2012). Appl. Catal., A.

[cit46] Zhu Y., Kong X., Li X., Ding G., Zhu Y., Li Y. (2014). ACS Catal..

[cit47] Guo X., Traitangwong A., Hu M., Zuo C., Meeyoo V., Peng Z., Li C. (2018). Energy Fuels.

[cit48] Ding J., Popa T., Tang J., Gasem K. A. M., Fan M., Zhong Q. (2017). Appl. Catal., B.

[cit49] Zhou H., Zhu W., Shi L., Liu H., Liu S., Ni Y., Liu Y., He Y., Xu S., Li L., Liu Z. (2016). J. Mol. Catal. A: Chem..

[cit50] Guo X., Peng Z., Hu M., Zuo C., Traitangwong A., Meeyoo V., Li C., Zhang S. (2018). Ind. Eng. Chem. Res..

[cit51] Moodley D. J., van de Loosdrecht J., Said A. M., Datye M. J., Overett A. K., Niemantsverdriet J. W. (2009). Appl. Catal., A.

[cit52] Alzate-Restrepo V., Hill J. M. (2008). Appl. Catal., A.

[cit53] Lin J., Zhao X., Cui Y., Zhang H., Liao D. (2012). Chem. Commun..

[cit54] Wen C., Cui Y., Dai W., Xie S., Fan K. (2013). Chem. Commun..

