# Supplementary material for: Highly active Ce, Y, La-modified Cu/SiO2 catalysts for hydrogenation of methyl acetate to ethanol
Source: RSC Adv. 2020 Feb 4;10(10):5590–603. doi: 10.1039/c9ra08780j (PMC9049221; doi:10.1039/c9ra08780j)
Supplement: RA-010-C9RA08780J-s001 [file RA-010-C9RA08780J-s001.pdf]

Electronic Supplementary Material (ESI) for RSC Advances.
This journal is © The Royal Society of Chemistry 2020

Supporting Information for

Highly active Ce, Y, La modified Cu/SiO_2 catalysts for hydrogenation of methyl acetate to ethanol

Zhiheng Ren,[abc] Muhammad Naeem Younis,[c] Chunshan Li,[cd*] Zengxi Li,[e] Xiangui Yang,[ab*] Gongying Wang,[ab]

[a] *Chengdu Institute of Organic Chemistry, Chinese Academy of Sciences, Chengdu, 610041, China. E-mail: yangxg@cioc.ac.cn.*

[b] *National Engineering Laboratory & Technology, University of Chinese Academy of Science, Beijing 101408, China.*

[c] *CAS Key Laboratory of Green Process and Engineering, State Key Laboratory of Multiphase Complex Systems, The National Key Laboratory of Clean and Efficient Coking Technology, Beijing Key Laboratory of Ionic Liquids Clean Process, Institute of Process Engineering, Chinese Academy of Sciences, Beijing 100190, PR China.*

[d] *Zhongke Langfang Institute of Process Engineering, Hebei Province, PR China E-mail: csli@ipe.ac.cn.*

[e] *School of Chemical Science, University of Chinese Academy of Sciences, Beijing 100049, People's Republic of China.*

Table S1. Product analysis from GC-MS.

Time/min	Product	Molecular Weight
1.32	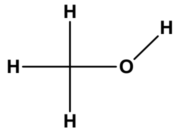	32
1.4	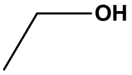	46
1.55	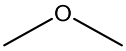	46
1.58	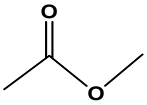	74
1.7	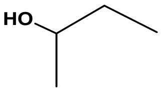	74
1.78	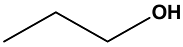	60
1.88	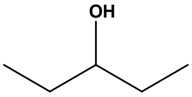	88
1.94	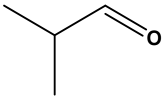	72
2.06	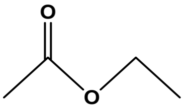	88
2.41	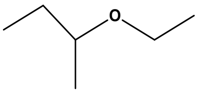	102
2.58	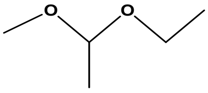	104

Reaction condition: T=280 ^{o}C, P=2.5MPa, GHSV=3000 h^{-1}, LHSV=1 h^{-1}.

Main Side reaction:

$$CH_3COOCH_3 + CH_3CH_2OH \rightarrow CH_3COOC_2H_5 + CH_3OH \quad (1)$$

$$CH_3CH_2OH \rightarrow CH_3CHO + H_2 \quad (2)$$

$$CH_3CH_2OH \rightarrow CH_3CH_2 + H_2O \quad (3)$$

$$CH_2CH_2 + H_2 \rightarrow CH_3CH_3 \quad (4)$$

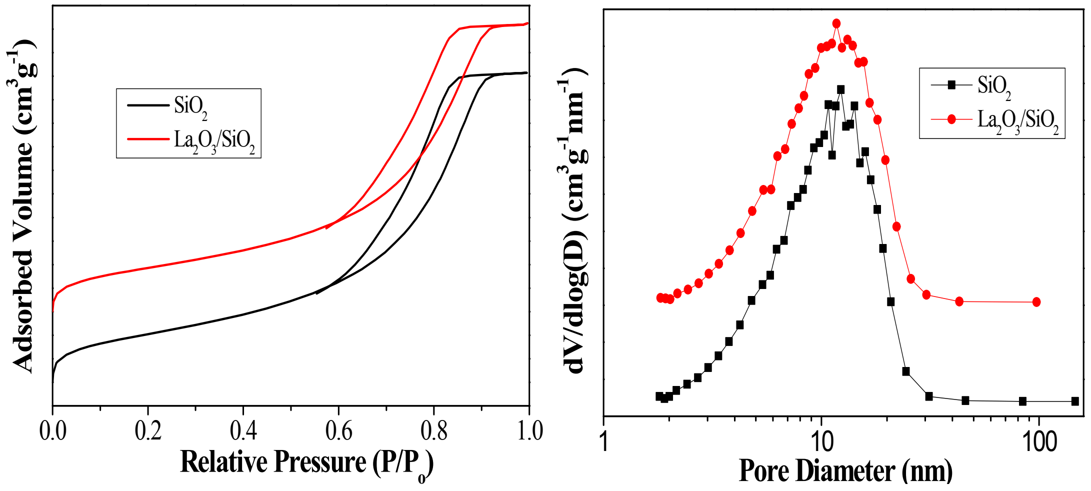

Fig. S1. N_2 adsorption-desorption isotherm and pore distribution of selected SiO_2 and La_2O_3/SiO_2.

Table S2. Grain size of the copper crystallite of different catalyst.

Catalyst	*2θ of 111Crystal face (°)*	d_{Cu} *(nm)*
Cu/SiO_2	43.4	4.5
Cu/SiO_2-5Ce	43.5	4.1
Cu/SiO_2-5Y	43.5	7.7
Cu/SiO_2-1La	43.5	3.6
Cu/SiO_2-5La	43.5	3.5
Cu/SiO_2-10La	43.5	7.1
Spent Cu/SiO_2	43.1	8.6
Spent Cu/SiO_2-5La	43.2	5.2

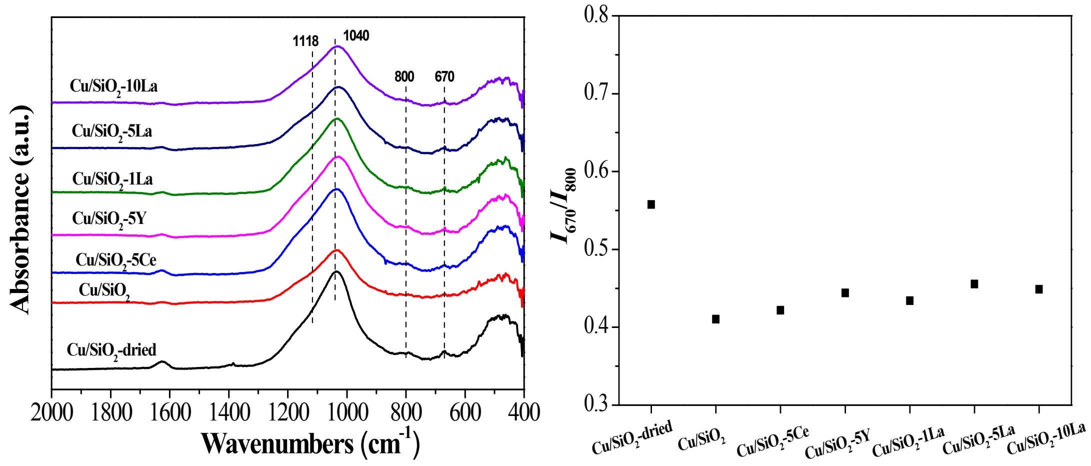

Fig. S2. FTIR spectra of $Cu-SiO_2$ and Cu/SiO_2-xM catalysts.

The IR bands of δ_{OH} = 670 cm^{-1} and υ_{SiO} = 1040 cm^{-1} could be suggested that the presence of copper phyllosilicate phase in all samples, while silica exhibits an asymmetric υ_{SiO} band at 1118 cm^{-1} and a symmetric υ_{SiO} band at 800 cm^{-1}. I_{670}/I_{800} is denoted the relative contents of copper phyllosilicate in different catalysts.[1-2] It could be found that the value of different calcined catalyst is almost the same, suggesting the amount of $Cu_2SiO_5(OH)_2$ in different catalyst has no big difference.

[1] T. Toupance, M. Kermarec, C. Louis, *J. Phys. Chem. B* 2000, **104**, 965–972.

[2] H. Yue, Y. Zhao, S. Zhao, B. Wang, X. Ma and J. Gong, *Nat. Commun.,* 2013, **4**, 2339–2345.

N_2O titration method

The copper surface area was calculated combined with the results of N_2O titration and Cu LMM XAES spectra according to previous report.[2-3] Typically, 50 mg of the calcined catalysts were reduced by the same H_2-TPR procedure described **in section 2.2** (denoted as total TPR). After cooling down to 60 ^{o}C, the reduced samples were exposed to a pure N_2O flow (60 mL/min, 1 h) to completely oxidize surface copper atoms into Cu_2O. Afterwards, the resulting surface oxidized samples underwent the second TPR run as the first one (denoted as surface TPR). The Cu dispersion (D_{Cu}), specific expose Cu surface area (S_{Cu}) were calculated by Eqs. (1) and Eqs. (2), by assuming 1.47×10^{19} copper atoms per square meter. Then the S_{Cu^+} (S_{Cu^0}) could be calculated combined the values of S_{Cu} and the Cu^+ (Cu^0) ratio from the results of Cu LMM XAES under the assumption that the Cu^+ ions and Cu^0 atoms occupy identical areas.

$$D_{Cu}(\%) = \frac{2\times \mathrm{H_2\ Consumption\ (surface\ TPR)}}{\mathrm{H_2\ Consumption\ (total\ TPR)}}\times 100 \qquad (1)$$

$$S_{Cu}(m^2g^{-1}Cu) = \frac{D_{Cu}\times N_{av}}{M_{\mathrm{Cu}}\times 1.46\times 10^{19}} \qquad (2)$$

Where N_{av}: Avogadro constant = 6.02×10^{23} (mol^{-1}); M_{Cu}: relative atomic mass =63.55 (g/mol).

[3] Y. Zhao, B. Shan, Y. Wang, J. Zhou, S. Wang and X. Ma, *Ind. Eng. Chem. Res.,* 2018, **57,** 4526–4534.

[4] P. Ai, M. Tan, P. Reubroycharoen, Y. Wang, X. Feng, G. Liu, G. Yang and N. Tsubaki, *Catal. Sci. Technol.*, 2018, **8**, 6441–6451.

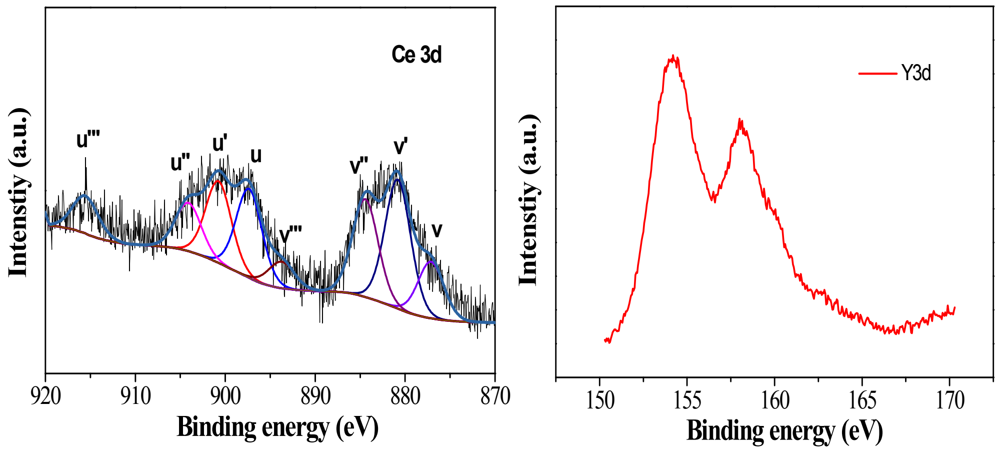

Fig. S3. XPS spectra of Ce 3d and Y 3d.

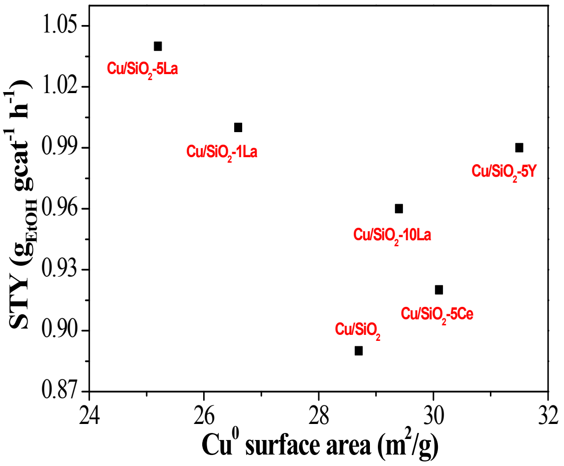

Fig. S4. Effects of Cu^0 surface area of Cu-SiO_2 and Cu/SiO_2-xM catalyst on the STY_{EtOH}.

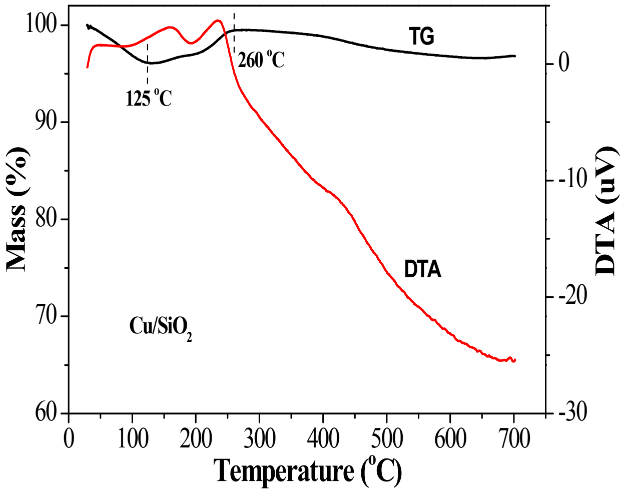

Fig. S5. TG and DTA analysis of spent Cu/SiO_2 after long-term stability test.

From the result of Figure S5, it could be found that there have three steps in the

whole stage. The first step before 125 °C is related to the weight loss of surface water. After that, the weight has increased might be due to the Cu^{+} species oxidation when exposed to the air. The third step after 260 °C is probably corresponding to the decomposition of CuO. From previous literatures, the carbon deposit would be oxidized at 200-400 °C. But from our results, it could be inferred that the formation of carbon deposition is not obvious.

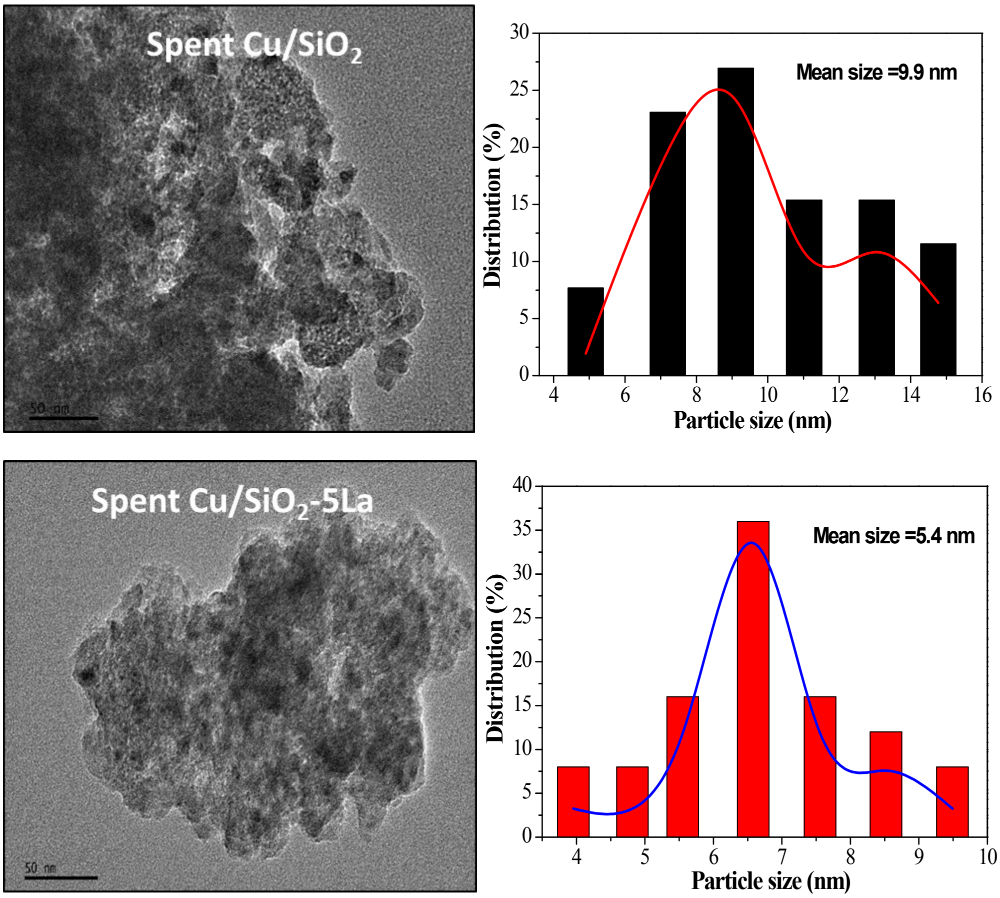

Fig. S6. TEM image of spent Cu/SiO_2 and $Cu\text{-}/SiO_2$-5La catalyst after long-term estimating.

Table S3. Summary of Cu-based catalysts for hydrogenation of MA to ethanol under different reaction conditions.

Catalyst	Pressure (Mpa)	LHSV (h^{-1})	H_2/MA	Reaction temperature (°C)	MA conversion (%)	Ref.
Cu/SiO_2-HP	2.5	2	15	250	86.8	This work
Cu/SiO_2-5La-HP	2.5	2	15	250	94.3	This work
Cu/SiO_2-AE	2	1	**40**	220	80.9	5
Cu/SiO_2-AE	**3**	2	**15**	250	83.7	6
Cu_9Zn_1/SiO_2	2	2	**20**	220	92	7
$Cu_{0.9}Zn_{0.1}$/SBA-15	2	2	**20**	220	85	8
Cu@CeO_2	2.5	**1**	**80**	215	99	9
Cu/9MgO-SiO_2	**3**	2	10	340	80.3	10
3Mn-Cu/SiO_2	**3**	2	15	250	96	11

HP: hydrolysis-precipitation method; AE: ammonia evaporation

[5] X. Dong, X. Ma, H. Xu and Q. Ge, *Catal. Sci. Technol.,* 2016, **6**, 4151–4158.

[6] Y. Zhang, C. Ye, C. Guo, C. Gan and X. Tong, *Chinese J Catal.,* 2018, **39**, 99–108.

[7] Y. Zhao, B. Shan, Y. Wang, J. Zhou, S. Wang and X. Ma, *Ind. Eng. Chem. Res.,* 2018, **57**, 4526–4534.

[8] Y. Wang, J Liao, J. Zhang, S. Wang, Y. Zhao and X. Ma, *AIChE J,* 2016, **63**, 2839–2849.

[9] Y. Wang, Y. Zhao, J. Lv and X. Ma, *ChemCatChem,* 2017, **9**, 2085–2090.

[10] H. Qin, C. Guo, C. Sun and J. Zhang, *J Mol. Catal. A,* 2015, **409**, 79–84.

[11] C. Ye, C. Guo, C. Sun and Y. Zhang, *RSC Adv.,* 2016, **6**, 113796–113802.
